# Molecular and Pathological Investigations of Selected Viral Neuropathogens in Rabies-Negative Brains of Cats and Dogs Revealed Neurotropism of Carnivore Protoparvovirus-1

**DOI:** 10.3389/fvets.2021.710701

**Published:** 2021-08-19

**Authors:** Sabrina Wahyu Wardhani, Boonyakorn Wongsakul, Tanit Kasantikul, Chutchai Piewbang, Somporn Techangamsuwan

**Affiliations:** ^1^The International Graduate Program of Veterinary Science and Technology, Faculty of Veterinary Science, Chulalongkorn University, Bangkok, Thailand; ^2^Animal Virome and Diagnostic Development Research Group, Faculty of Veterinary Science, Chulalongkorn University, Bangkok, Thailand; ^3^Department of Animal Diagnosis and Investigation, Queen Saovabha Memorial Institute, The Thai Red Cross Society, Bangkok, Thailand; ^4^Clemson Veterinary Diagnostic Center, Clemson University, Columbia, SC, United States; ^5^Department of Pathology, Faculty of Veterinary Science, Chulalongkorn University, Bangkok, Thailand

**Keywords:** brain, cat, CPPV-1, dog, neurological disorder, viral infection

## Abstract

Throughout the year, the Thai Red Cross Society (TRCS), Bangkok, Thailand, received more than 100 animals that died of suspected rabies due to neurological clinical signs. Concerning the role of viral infection in the brain in the outcome of neurological diseases in cats and dogs, a comprehensive study was conducted of 107 brain samples of cats and dogs submitted to the TRCS from August 2019 to August 2020. Selective molecular screening using conventional polymerase chain reaction (PCR) and reverse transcription PCR targeting nine viral pathogens was employed in addition to histopathological investigations. The results showed that *carnivore protoparvovirus-1* (CPPV-1) was detected in 18.69% of the cats and dogs sampled (20/107). These results were found in young and old animals; the brain tissue did not show any pathological changes suggesting encephalitis or cerebellar hypoplasia. In addition, *feline calicivirus, feline alphaherpesvirus-1, feline coronavirus*, and *canine distemper virus* were also detected, providing a broader range of potential viral infections to consider in the clinical manifestation of neurological disorders in companion animals. The detection of all pathogens was confirmed by the localization of each viral antigen in various resident brain cells using immunohistochemistry. A unique L582S amino acid substitution of the non-structural protein 1 gene coding sequence, speculated to be associated with the neurotropism of CPPV-1 in cats and dogs, was not evident. In conclusion, this study revealed a noteworthy neurotropism of CPPV-1 in both cats and dogs without neurological lesions.

## Introduction

Neurological disorders are one of the most common health issues presented by cats and dogs in animal hospitals, caused by many underlying conditions (1). Among the underlying causes of the neurological disorder, inflammation of the brain or encephalitis is responsible for up to one-third of the overall cases. One of the causes of encephalitis is viral infection, detected in a high number of infectious encephalitis cases. In addition, viral infection is also suspected in many cases of encephalitis with unknown origin. A specific pathogen was not detected, but the histopathological changes in the brain show a non-suppurative inflammatory reaction. This suggests an underlying viral infection (1–3). Clinical data from two feline referral hospitals in the UK revealed that brain infection was responsible for ~30–45% of the overall neurological disorder cases. Most of the cases showed histopathological changes suggestive of viral infection (3). Similarly, viral infection was also believed to be the underlying cause in many encephalitis cases of unknown origin in dogs (1). This highlights the significant role of viral infection in central nervous system (CNS) diseases in cats and dogs, yet much remains to be revealed.

Previously, some viruses, such as coronavirus, herpesvirus, paramyxovirus, and rhabdovirus, were shown to cause encephalitis in cats and dogs with various clinical manifestations (4). Other viruses, including adenovirus and flavivirus, have been detected in encephalitis cases without other accompanying infections (2, 4–6). *Carnivore protoparvovirus 1* (CPPV-1) without cerebellar hypoplasia infection in the brain of companion animals has also been reported in various clinical cases of CNS. This fact emphasizes the unresolved role of CPPV-1 in the neurological disorders in cats and dogs (7, 8).

Even though evidence of the virus in the cat's and dog's brains is growing, there are still limitations about the number of samples and identifiable viral pathogens from clinical cases in animal hospitals. Attention has only focused on non-suppurative encephalitis cases confirmed by histopathological examination. Furthermore, basic information about virus detection in cats and dogs suspected of encephalitis in Thailand is unclear. Therefore, in this study, we conducted selective viral molecular screening of the brain in rabies-negative cats and dogs that presented neurological signs before death and were submitted to the Thai Red Cross Society (TRCS). Various viral neuropathogens were detected in the rabies-negative brains of those animals. Moreover, we examined the localization of each viral pathogens using immunohistochemistry (IHC) with an additional focus on the genetic characterization and diversity of the CPPV-1.

## Materials and Methods

### Animals and Sample Collection

A total of 107 brain samples from rabies-negative cats (*n* = 56) and dogs (*n* = 51) submitted to the TRCS, Bangkok, Thailand, from August 2019 to August 2020, were enrolled in this study. Brain samples were collected only from cats and dogs that were negative for rabies based on the direct fluorescent antibody test (dFAT) and confirmed by the mouse inoculation test (MIT). Brains that tested positive for rabies by dFAT or MIT were excluded from the study. The age of the cats and dogs ranged from 2 months to 19 years. Following the American Animal Hospital Association (AAHA), the animals were classified by age. Those classifications, along with their number and percent of the study sample, are as follows. Cats were classified as a kitten (birth to 6 months, 17.76%, *n* = 19), junior (6 months to 2 years, 8.41%, *n* = 9), adult (2–10 years, 23.36%, *n* = 25), and senior (more than 10 years, 2.8%, *n* = 3) (9). Dogs were classified by age as a puppy (birth to 6 months, 10.28%, *n* = 11), junior (6 months to 2 years, 5.61%, *n* = 6), adult (2–6 years, 24.3%, *n* = 26), and senior (more than 6 years, 7.48%, *n* = 8) (10).

Sample collection was limited to the carcass received by the TRCS. Therefore, only a brief history of clinical presentation and signalments could be collected without a prior examination by veterinarians. Neurological signs including seizure, paralysis, blindness, difficulty swallowing, and muscle spasms were observed in 40.19% of animals (*n* = 43). Non-specific clinical signs, such as anorexia, lethargy, vomiting, diarrhea, epistaxis, nasal discharge, aggression, and depression, were recorded in 50.47% of the animals (*n* = 54). In 9.34% (*n* = 10), clinical signs were not evident.

Brain tissues, consisting of cerebrum, cerebellum, and brainstem, were collected from fresh samples and immersed in 10% neutral buffered formalin following routine rabies-testing. Fresh tissues were stored at −80°C until further processed, while formalin-fixed tissues were routinely processed for histopathological examination. All protocols in this study were conducted under the approval of the Institutional Biosafety Committee of Chulalongkorn University (IBC No. 2031002) and the Queen Saovabha Memorial Institute Animal Care and Use Committee (Protocol No. QSMI-ACUC-01-2020).

### Molecular Detection and Genomic Analysis

Approximately 20–30 mg of fresh brain tissue samples were submitted for total viral nucleic acid extraction using a commercial viral DNA/RNA extraction kit II (Geneaid, New Taipei City, Taiwan) following the manufacturer's protocol. The quality and quantity of the extracted viral nucleic acids were determined using a spectrophotometer (NanoDrop, Thermo Scientific™, Waltham, MA, USA) *via* the 260/280 ratio absorbance.

All viral nucleic acid extracts were screened using different sets of consensus primer with broad reactivity to viruses in the families *Coronaviridae* (11), *Flaviviridae* (12), *Herpesviridae* (13), and *Paramyxoviridae* (14) and the genus *Protoparvovirus* (15). For nucleic acids derived from the cat samples, additional PCR screenings for *feline calicivirus* (FCV) (16) and *feline bocavirus* (FBoV) (17) were conducted. For nucleic acids derived from dog samples, additional screenings for *canine adenovirus* (CAdV) (18, 19) and *canine bocavirus* (CBoV) (17) were conducted. Selective viral screenings were performed using conventional PCR (cPCR) employing GoTaq Green Master Mix (Promega, USA) or cRT-PCR utilizing Qiagen OneStep RT-PCR kit (Qiagen GmbH, Hilden, Germany) (Supplementary Table 1).

The PCR products were run on a 2% agarose gel with 5% ethidium bromide in gel staining and visualized using a UV illuminator. All positive amplified products from electrophoresis gel were cut and extracted using NucleoSpin® Gel and PCR Clean-up kit (NucleoSpin®, Germany) according to the manufacturer's instructions. Purified PCR products were submitted for bidirectional sequencing to Macrogen (Macrogen Co., Ltd., Korea). The sequencing results were analyzed and edited where necessary using BioEdit 7.0 software (https://bioedit.software.informer.com/7.2/). The resulting sequences were then compared with the previously published sequences available in the GenBank database using the Basic Local Alignment Search Tool (BLAST) program from the National Center for Biotechnology Information (NCBI) (https://blast.ncbi.nlm.nih.gov/Blast.cgi).

### Whole-Genome Sequencing and Analysis of CPPV-1

Eight representative samples positive for CPPV-1 screenings were subjected to additional cPCR assays to obtain the complete genome sequences of CPPV-1 using multiple sets of primer (Supplementary Table 2) derived from previous studies (15, 20). For further analysis of the complete parvovirus genome, sequences were aligned with Multiple Alignment using Fast Fourier Transform (MAFFT) version 7 (https://mafft.cbrc.jp/alignment/server/). Subsequently, a phylogenetic tree was constructed using Molecular Evolutionary Genetics Analysis (MEGA) 7.0 software (http://www.megasoftware.net/) with the maximum likelihood (ML) method and bootstrap analysis with 1,000 replicates. The model used for phylogenetic tree construction was selected from the lowest Bayesian information criterion (BIC) number based on the find-best-fit model algorithm embedded in the MEGA 7 software.

### Histopathological Examination

Formalin-fixed paraffin-embedded (FFPE) tissue from each brain sample was utilized for routine histology. Slides were individually examined by a Thai board-certified veterinary pathologist (ST) and an American board-certified veterinary pathologist (TK). The criteria for examination included perivascular cuffing, neuronophagia, neuronal degeneration, neuronal necrosis, gliosis, and evidence of inclusion bodies. The histopathological findings were classified into four groups: (1) “no significant changes” for brains tissues that showed no change in the histological structure, (2) “mild” with ≤ 25% affected area in the brain, (3) “moderate” with 26–50% affected area in the brain, and (4) “severe” with ≥50% affected area in the brain.

### Immunohistochemistry

IHC was conducted to confirm the PCR results and determine the cellular tropism and localization pattern of each virus detected in the initial PCR screening. Horseradish-peroxidase IHC using the Dako REAL EnVision Detection System (Dako, Glostrup, Denmark) was used to detect the positive antigen–antibody signal. Consistent with the PCR screening results, IHC was conducted for five viruses, including *feline coronavirus* (FCoV), *feline alphaherpesvirus-1* (FeHV-1), *canine distemper virus* (CDV), CPPV-1, and FCV.

FFPE tissues were cut into 4-μm sections and placed on positively charged slides. Following deparaffinization, the slides were triple washed with 1 × phosphate-buffered saline (PBS) prior to the antigen retrieval process (Supplementary Table 3). The slides were then immersed in 3% (v/v) hydrogen peroxide (H_2_O_2_) for 15 min to block endogenous peroxidase activity and 5% (w/v) skim milk for 1 h at 37°C to block non-specific reactions. Subsequently, a primary antibody targeting each potential virus (Supplementary Table 3) was applied, and slides were incubated at 4°C overnight. Later, an anti-mouse, anti-rabbit secondary antibody (Dako REAL EnVision Detection System) was used. The antigen–antibody complex was then labeled with 3,3′-diaminobenzidine (DAB), counterstained with hematoxylin, and then examined under a light microscope.

IHC universal IgG negative control (Enzo, ADI-950-231, USA) was used as the secondary antibody for negative control. Slides from cases that previously tested positive for each targeted virus *via* PCR were employed for positive control. The examination was done in all areas of the slides, and results were analyzed descriptively to show cell tropism and viral localization in the brain.

## Results

### Molecular Detection of Viral Pathogens in the Brain

cPCR/RT-PCR employed in this study detected viral pathogens in 18/56 (32.14%) cat brain samples and 9/51 (17.65%) dog brain samples. From all PCR panels in the molecular screening, positive results were achieved from five panels for *Protoparvovirus, Paramyxoviridae, Herpesviridae, Coronaviridae*, and FCV. To confirm the specific viral pathogens detected from the brain, all positive samples were subjected to bidirectional Sanger sequencing. The obtained sequences were compared to the available sequences published in the GenBank database using BLASTn analysis. Five viral pathogens were detected, including CPPV-1 [specifically the feline parvovirus (FPV) and canine parvovirus (CPV)], FCV, FCoV, FeHV-1, and CDV.

CPPV-1 was the most common viral pathogen detected in the brain of both cats and dogs. It was detected in young, adult, and senior individuals. FPV was detected in 25% of the overall samples retrieved from cats (*n* = 14), including five kittens, two juniors, six adults, and one senior. In comparison, CPV was found in six dog brain samples (11.76%), including five puppies and one adult (Table 1).

**Table 1:** Positive cases from molecular screening.

**Case number^**a**^**	**Viral pathogen^**b**^**		**Host**	**Age^**c**^**
B29	FCV		Cat	5 mth
B31	FPV		Cat	12 yr
B32	FPV		Cat	>2 yr
B34^a^	FPV		Cat	4 mth
B57	FPV		Cat	5 mth
B59^a^	FPV		Cat	>2 yr
B60	FeHV-1		Cat	4 yr
B63^a^	CPV		Dog	3 mth
B64	FPV		Cat	3 mth
B69	FPV		Cat	4 yr
B74	FPV		Cat	4 mth
B75	FPV		Cat	2 yr
B82	FPV		Cat	4 mth
B86	FPV		Cat	>2 yr
B88^a^	FPV		Cat	>2 yr
B90^a^	CPV		Dog	3 mth
B94	FPV		Cat	2 yr
B100	FCoV and FeHV-1		Cat	2 mth
B102^a^	CPV		Dog	>2 yr
B110	CPV		Dog	4 mth
B111^a^	CPV		Dog	3–4 mth
B112^a^	CPV		Dog	3–4 mth
B118	CDV		Dog	4 mth
B127	FPV		Cat	>2 yr
B132	CDV		Dog	10 yr
B135	FCV		Cat	1.5 yr
B137	CDV		Dog	7 yr

a *Selected cases for whole-genome sequencing of Carnivore protoparvovirus 1.*

b *CDV, canine distemper virus; CPV, canine parvovirus; FCoV, feline coronavirus; FCV, feline calicivirus; FeHV-1, feline herpesvirus-1; FPV, feline parvovirus.*

c *yr, year; mth, month*.

In addition to CPPV-1 detection, two samples from cats (3.57%) tested positive for FCV; two other samples (3.57%) tested positive for FeHV-1; and one sample (1.79%) tested positive for FcoV. Three samples from dogs (5.89%) tested positive for CDV. Dual detection of FCoV and FeHV-1 was found in one cat (case no. B100), while other cases revealed only one detection. FCV brain infections were found in one kitten and one adult cat. FeHV-1 brain infections were observed in one kitten and one adult cat. CDV was detected from the brain of one puppy and two senior dogs (Table 1).

### Whole-Genome and Deduced Amino Acid Analysis of CPPV-1

In the initial PCR screening for parvovirus, partial VP2 gene sequences were obtained from all 20 parvovirus-positive samples. BLASTn analysis based on the partial VP2 sequences revealed 14 sequences retrieved from cats. They showed 99.02–100% nucleotide identity to FPV strains from Thailand, Italy, South Korea, and Russia, with the highest identity percentage (100%) to FPV strain from Thailand (accession no. MN127779). All six sequences retrieved from dogs shared 99.09–99.93% nucleotide identity to CPV-2c strains from China, the highest identity percentage (99.93%) to CPV-2c isolate CPV-AHhf1 (accession no. MT010564) (Supplementary Table 4).

Whole-genome sequencing was conducted for eight representative cases exhibiting positive CPPV-1 detection. These were selected based on histopathological changes, including cases with and without inflammatory reaction. These eight samples were designated as (1) CPV 2c/TRC-B63/Thailand/2020; (2) CPV 2c/TRC-B90/Thailand/2020; (3) CPV 2c/TRC-B102/Thailand/2020; (4) CPV 2c/TRC-B111/Thailand/2020; (5) CPV 2c/TRC-B112/Thailand/2020; (6) FPV/TRC-B34/Thailand/2020; (7) FPV/TRC-B59/Thailand/2020; and (8) FPV/TRC-B88/Thailand/2020 (accession no. MW589466–MW589473).

**Figure 1:**
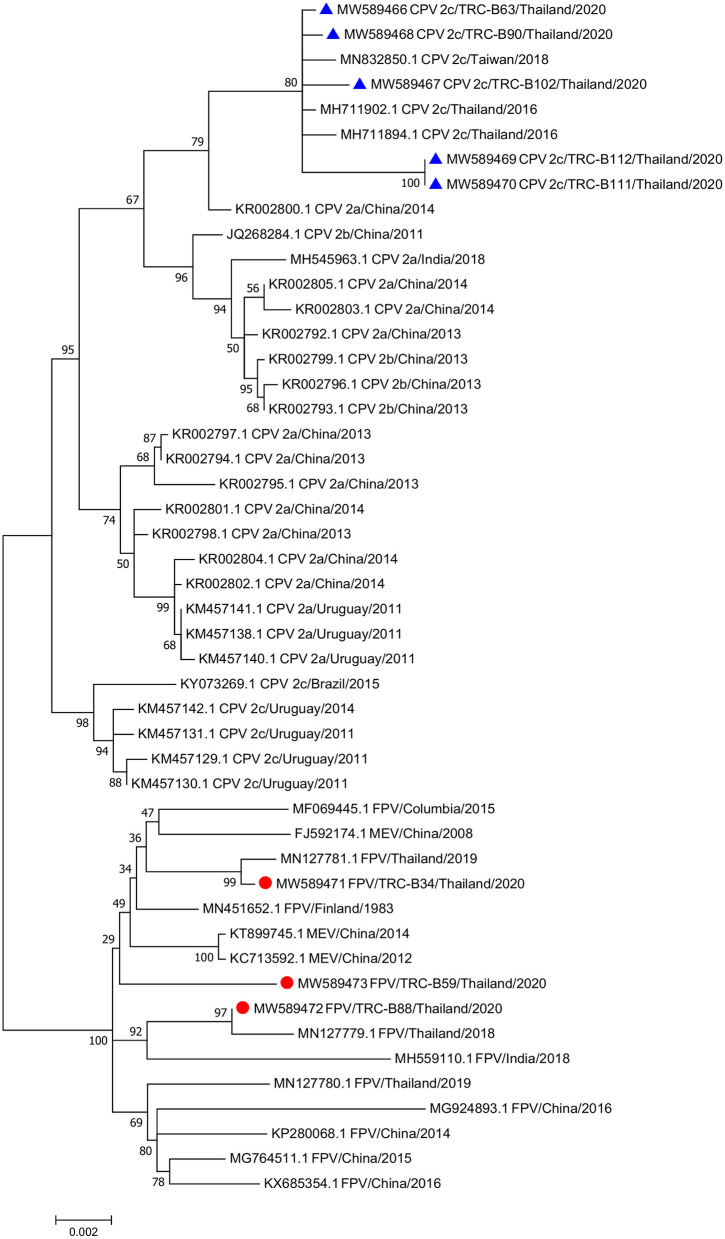
Phylogenetic tree of the complete genome sequences of *feline parvovirus* (FPV) and *canine parvovirus* (CPV) obtained from three cats and five dogs in this study and the available sequences from GenBank. Sequences from cats are labeled as (•) while sequences from dogs are labeled as (▴).

A maximum likelihood phylogenetic tree was constructed from the FPV and CPV nucleotide sequences obtained from this study with the addition of available reference sequences in the GenBank database (Supplementary Table 5). The Hasegawa–Kishino–Yano (HKY) model was implemented based on the best-fit substitution model calculation with the lowest BIC number. The phylogenetic tree showed that the eight complete genome sequences were divided into two distinct clusters; all three sequences from cats (MW589471–MW589473) were grouped in the FPV cluster, while the other five complete sequences from dogs (MW589466–MW589470) were separated into the CPV cluster. The phylogram demonstrated a close relationship between the CPV-2c obtained from this study and a previously reported CPV-2c in Thailand (MH711902 and MH711894), and a reported CPV-2c sequence from Taiwan (MN832850) (Figure 1).

Furthermore, the deduced amino acid alignment analysis from the eight complete genome sequences of cats and dogs showed no unique mutation in either the NS or VP gene compared to reference sequences (data not shown).

### Histopathological Findings and Localization of Viral Antigen

#### CPPV-1 Localization in Brains

**Figure 2:**
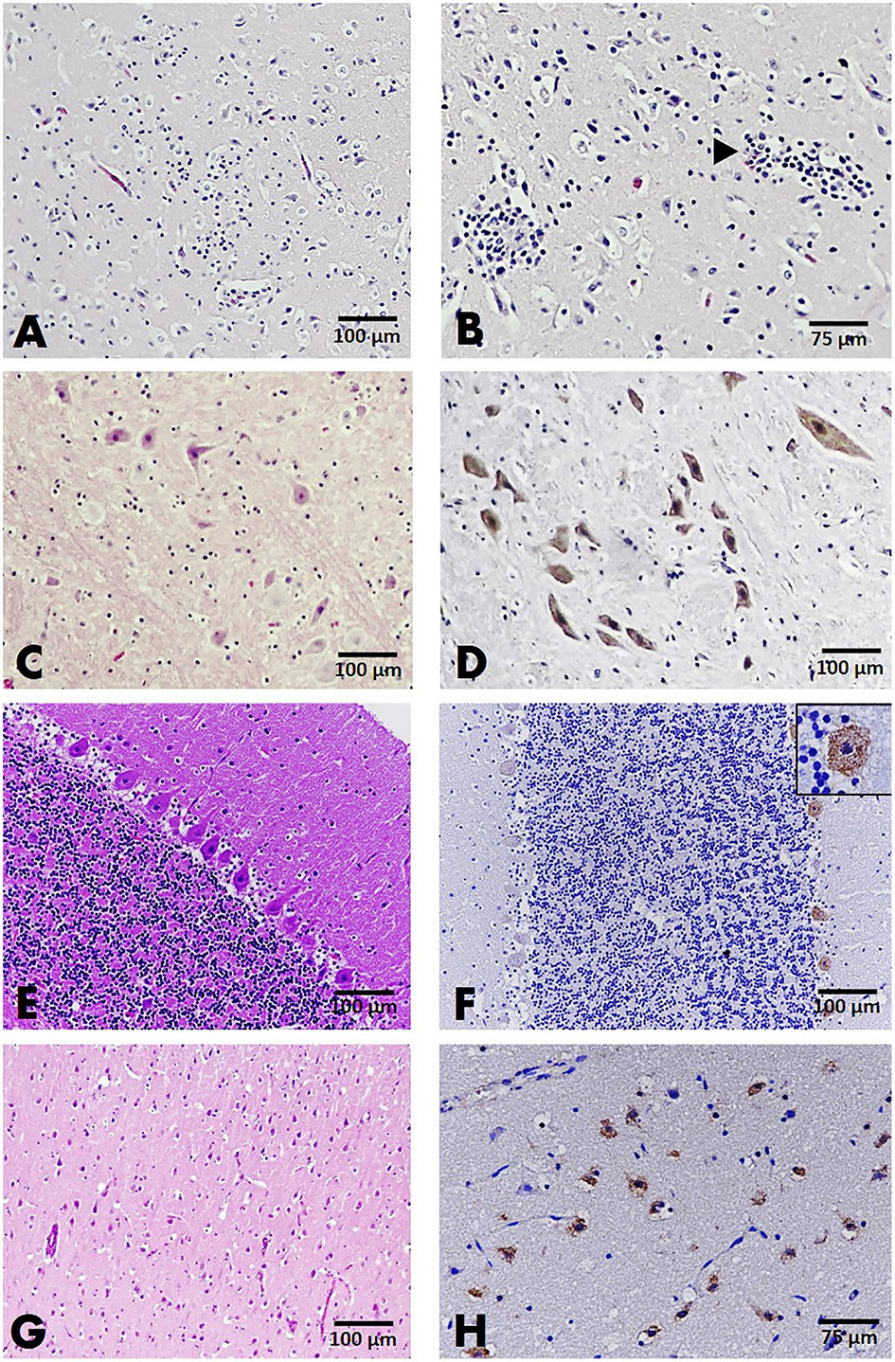
Brain, cat **(A–F)**, and dog **(G,H)**. **(A,B)** The cerebral cortex of cat no. B32 presented gliosis **(A)** and two to three layers of perivascular cuffing (arrowhead) **(B)**, HandE staining. **(C)** Dark neurons were evident in the gray matter of the brain stem, HandE staining. **(D)** Immunoreactivity to parvovirus antigen in large neurons comprising the brain stem, IHC for parvovirus. **(E)** Section of the cerebellum showing no significant change (HandE staining), but demonstrating positive immunostaining to the parvovirus antigen in the cytoplasm of Purkinje cells (inset) composing the cerebellar cortex **(F)**. **(G)** Section of the cerebral cortex from a dog showing no significant changes in the resident cells, HandE staining. **(H)**. Parvovirus-antigen-positive staining was demonstrated in the small neurons comprising the cerebral cortex. IHC for parvovirus.

A total of 107 brain sections were examined, with results from cases with positive viral detection in the brain emphasized. Histopathological examination on parvovirus-positive cases revealed that non-suppurative encephalitis or meningitis was found in only two cats (case nos. B32 and B34) in 20 brain sections from affected cats and dogs. In case no. B32, severe perivascular cuffing, characterized by two to three layers of perivascular lymphocytic infiltration, multifocal gliosis, degenerative neurons, and satellitosis were observed in the cerebrum and cerebellum (Figures 2A,B). In other cases, alterations of the resident brain cells, such as gliosis and dark neurons, were observed without inflammatory reaction (Figure 2C). Noteworthy was the greater number of brain sections from parvovirus PCR-positive cases; no significant histopathological changes were observed (Figures 2E,G).

IHC assays were conducted to localize the parvovirus antigen in seven representative cases, consisting of four cat and three dog brain sections. Immunoreactivity to the parvovirus antigen was found in all representative cases regardless of histopathological changes in the brain tissue. This finding was in contrast to the negative control slides that showed no immunoreactivity to the parvovirus antigen. Four brain sections from cats (case nos. B31; B21; B34; and B64) demonstrated positive immunostaining in the neurons composing cerebral gray matter and white matter. Immunoreactivity was also observed in large neurons of the brain stem and cerebellar Purkinje neurons (Figures 2D,F). In three brain sections of affected dogs (case nos. B90; B111; and B112), localization was observed in only the small neurons of the cerebral cortex (Figure 2H).

#### CDV Localization in Brains

**Figure 3:**
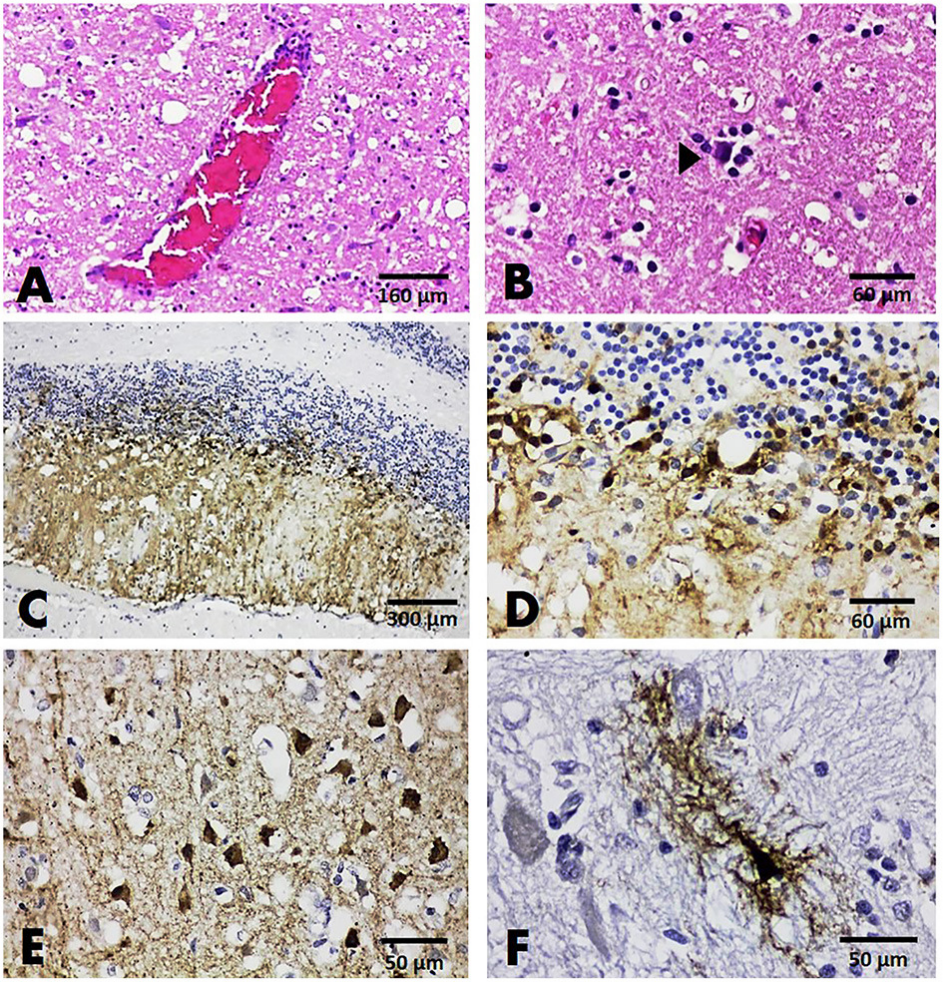
Brain, dog. HandE sections showing mild perivascular cuffing **(A)** and satellitosis (arrowhead) **(B)**. IHC slides showing positive immunoreactivity to the CDV antigen in the cells comprising cerebellar gray matter **(C,D)**, in small neurons **(E)**, and in the glial cells resembling astrocytes **(F)** of the cerebral cortex.

Histopathologically, among all three brain sections from dogs tested positive for CDV, non-suppurative encephalitis was presented in only one case (case no. B118). Two other cases (case nos. B132 and B137) showed only satellitosis and no significant changes, respectively (Figures 3A,B). No inclusion body was observed in any affected dogs. Immunoreactivity to the CDV antigen was demonstrated in all cases. In case no. B118, the immunolabeling of the CDV antigen was shown in small circular neurons composing the granular layer and the axonal and dendritic processes that breach the molecular layer (Figures 3C,D). A different localization pattern of the CDV antigen was evident in two other cases. In those cases, immunoreactivity to the CDV antigen was demonstrated in neurons and glial cells resembling astrocytes of the cerebrum (Figures 3E,F).

#### FCoV and FeHV-1 Localization in Brains

**Figure 4:**
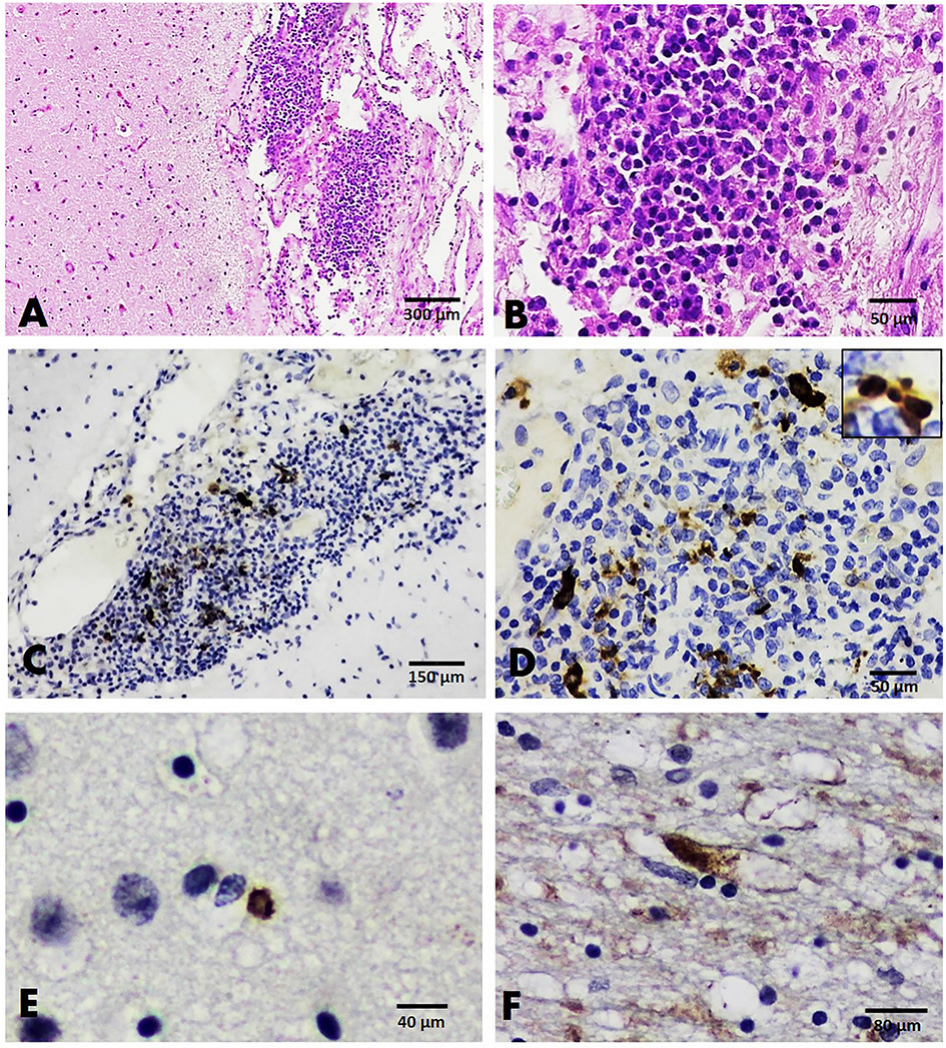
Brain, cat no. B100. **(A,B)** HandE sections from the FCoV-infected cat revealed the infiltration of inflammatory cells, primarily lymphocytes, and plasma cells, in the meningeal lining of the cerebrum **(A,B)**. **(C,D)** IHC staining with double detection of FCoV and FeHV-1 in the brain. Immunoreactivity to FCoV antigen was demonstrated by the inflammatory cells **(C,D)**. **(E,F)** Positive immunostaining to the FeHV-1 antigen was expressed by resident cells comprising the gray **(E)** and white matter of the cerebrum **(F)**.

A histopathological examination of a brain section from cat no. B100 (with dual detection of FCoV and FeHV-1) revealed severe non-suppurative meningitis. The meningitis was characterized by multifocal infiltration of inflammatory cells consisting of lymphocytes, plasma cells, and macrophages in the meningeal lining of the cerebrum and mild neuronophagia in the brain parenchyma (Figures 4A,B). Another brain section from a cat that tested positive for FeHV-1 (case no. B60) revealed no significant histopathological changes. Localization of the FCoV and FeHV-1 antigens was done in one representative brain section from cat no. B100. Immunolabeling to the FCoV antigen was explicitly found in the cytoplasm and nucleus of macrophages and lymphocytes composing the inflamed area of the cerebral meningeal layer (Figures 4C,D). Localization of FeHV-1 was demonstrated predominantly in resident cells comprising the cerebral gray and white matter (Figures 4E,F).

#### FCV Localization in Brains

**Figure 5:**
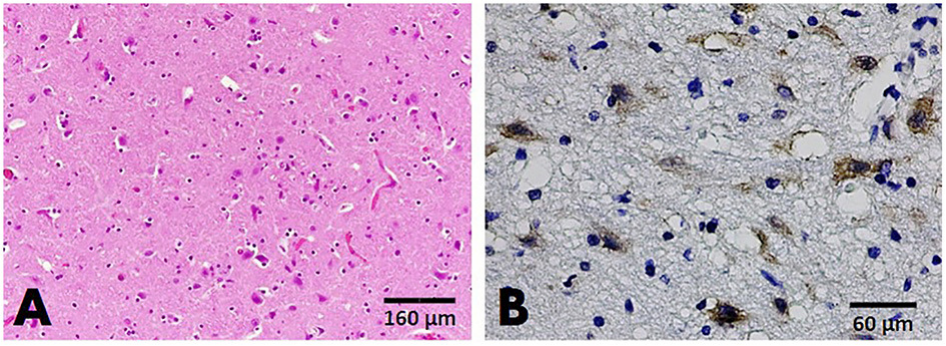
Brain, cat no. B135. **(A)** HandE staining showing mild satellitosis in the gray matter of the brain stem. **(B)** IHC slides showing positive immunoreactivity to the FCV antigen in small neurons comprising the cerebral cortex.

Examination of the histological sections of FCV-positive cats revealed mild satellitosis and dark neurons in the brain stem of cat no. B135 (Figure 5A), while cat no. B29 exhibited no significant changes. Positive immunoreactivity to the FCV viral antigen was expressed in both cats. Immunolabeling signals were seen in the small pyramidal neurons comprising the cerebral cortex and large neurons of the brain stem (Figure 5B).

In addition, out of the 107 brains subjected to histopathological examination in this study, non-suppurative encephalitis was present in 10 brains (9.35%). Among them, viral infection was detected in only two cases (case no. B32 with a FPV-infected cat and B118 with a CDV-infected dog). By comparison, in eight cases, cPCR/RT-PCR showed negative results for each specific virus included in this study. However, in one dog with negative results in all PCR panels for selective molecular screening, mild perivascular cuffing with the addition of an intracytoplasmic eosinophilic inclusion body inside the Purkinje cells of the cerebellar cortex was observed (Supplementary Figure 1). The summary of histopathological changes and IHC analysis of the representative sections from positive PCR samples are presented in Supplementary Table 6.

## Discussion

In recent decades, there has been growing evidence of viral infections in the brains of cats and dogs that exhibited clinical signs of neurological disorders. These findings suggest the essential role of viral infection in the outcome of neurological disorders in companion animals (4–6, 21). In this study, we aimed to investigate the occurrence of viral infections in the brains of cats and dogs that presented neurological clinical signs. To accomplish this, we conducted histopathological investigations of selected neuropathogens from the brains of cats and dogs previously suspected of rabies by their clinical signs but later proven to be rabies-negative.

Several pathogens, including CPV, CDV, FPV, FCV, FcoV, and FeHV-1, were detected with selective molecular screening using cPCR/RT-PCR. Detection was then confirmed by the localization of each viral antigen in the brain tissue by IHC. Findings in this study revealed that the most commonly detected pathogen in the brain of both cats and dogs (18.69%) was CPPV-1, including the highly contagious FPV and CPV. Previously, CPPV-1 infection in cats and dogs had been associated primarily with outcomes of life-threatening enteric disease and panleukopenia, owing to their restricted tropism in highly dividing cells (21). However, during the gestation period, the infection of FPV and CPV was observed to cause congenital defects affecting the developing cerebellum and myocardium. This led to cerebellar hypoplasia in cats and viral myocarditis in dogs (22).

Although the occurrence of viral-induced cerebellar hypoplasia was less likely in dogs than cats, a study by Schatzberg et al. reported detection of CPV in the brains of two dogs with cerebellar hypoplasia. This finding suggested the possibility of parvoviral etiology in canine cerebellar hypoplasia (23). Interestingly, our results showed no pathological changes suggesting cerebellar hypoplasia in 20 affected cats and dogs, implying that CPPV-1 infection in the brains of cats and dogs might be associated with CNS diseases other than cerebellar hypoplasia. These results support the findings of previous studies that reported CNS manifestation of CPPV-1 in cats and dogs associated with an outcome of neurodegeneration and leukoencephalopathy (7, 8). The mechanism that serves the infection of CPPV-1 outside the Purkinje layer of the cerebellum has yet to be elucidated. Early postulates claimed that neurons, unlike other cells in the body, are terminally differentiated in the adult nervous system. Therefore, they do not provide the appropriate requirement for parvovirus replication (24). Recent reports, however, highlight a possible mechanism whereby CPPV-1 causes the cell cycle to arrest in the S-phase, which is similar to the mechanism of another type of parvovirus, the minute virus of mice (MVM). This virus causes cell cycle arrest by promoting the DNA damage response (DDR) (25). A unique L582S amino acid substitution of the NS1 gene-coding sequence has also been speculated to be associated with the neurotropism of CPPV-1 in cats and dogs (21, 26), However, this unique L582S substitution was not found in any of the eight whole-genome sequences obtained in this study.

In addition to its high detection rate, there were two prominent findings on the detection of CPPV-1 in the brain of cats and dogs. First, the detection was not limited to young animals; they were also found in adult and senior individuals. Second, the CPPV-1 antigen was localized without the brain sections showing any histopathologic features of encephalitis or encephalopathy. These findings are similar to those of a study by Skuja et al. that reported the detection of another type of parvovirus, the human parvovirus B19 (B19V), in post-mortem brain tissues of the elderly, with and without encephalopathy. This finding supported evidence of a well-known B19V characteristic, the ability to persist in a latent state after first exposure (27). The potential of CPPV-1 to establish persistent infection in companion animals has never been described before. However, because our findings demonstrated the localization of the parvovirus antigen in the neurons of cats and dogs without any pathological changes in the brain tissue, it was intriguing to propose another possible mechanism of CPPV-1 infection in the brain. That is, it may exist in a quiescent state after first exposure in young animals. In this study, however, the ability to detect the absence of degenerative and inflammatory processes was limited because examinations were histopathological only. Thus, further investigation is needed to confirm the actual absence of degenerative and inflammatory processes in the brain of cats and dogs with the CPPV-1 infection.

In addition to FPV, FeHV-1 was detected in two cats' brains with robust localization of the viral antigen in neurons and resident cells comprising the cerebrum. Despite the nature of herpesvirus as a neurotropic virus, brain infection of herpesvirus in cats and dogs is a rare outcome compared to that of horses, pigs, and bovines (28). Reports of FeHV-1 infection in cats' brains are minimal, and we did not find any data regarding the localization of the FeHV-1 viral antigen in the brain during our literature review. Our findings, therefore, provide new information regarding the localization of FeHV-1 in the brains of cats. One case that was positive for FeHV-1 revealed coinfection with FCoV. Immunoreactivity to the FCoV antigen was limited to inflammatory cells of the meningeal layer, similar to that described in previous reports (29).

FCV was detected in the brain of two cats, and the viral antigen was localized to neurons of the cerebrum and the brain stem. FCV is not grouped into neurotropic viruses, but infection from this virus in the brain of a cat was reported from a single case of virulent systemic disease (VSD) with the localization of the viral antigen in the endothelial lining of the cerebellum, lung, and liver (30).

CDV was also detected in three dogs, and immunolabeling to the CDV antigen was evident in cells comprising the cerebrum, cerebellum (including Purkinje cells), neurons, and glial cells resembling astrocytes (31).

Finally, of the 10 cases that exhibited non-suppurative encephalitis, viral infection was detected in only two samples, while in eight samples, the underlying infectious agent was not detected. Non-suppurative encephalitis, however, was suggested as the underlying viral infection most of the time. Therefore, the possibility of infection from other viruses included in this study cannot be excluded. Protozoa such as *Toxoplasma gondii* might also elicit non-suppurative encephalitis in cats and dogs, but the occurrence is less reported (32). Moreover, viral infection was strongly suggested in one case that exhibited intracytoplasmic inclusion bodies in the Purkinje cells of the cerebellum. However, other underlying conditions such as neurogenerative diseases in aged animals may also form inclusion bodies. More advanced techniques such as transmission electron microscopy (TEM) should be employed in further studies to reveal the ultrastructure of the inclusion bodies.

In conclusion, this study revealed the detection of FPV, FcoV, FCV, FeHV-1, CPV, and CDV from cats and dogs previously suspected of rabies infection and presented various signs of neurological disorder. Thus, we provided a broader range of possible viral infections that should be considered in the clinical manifestation of neurological disorders in companion animals. In our findings, FPV and CPV were the most commonly detected pathogens from the brain of cats and dogs. These two viruses should be highlighted because they demonstrate distinct manifestations.

## Data Availability Statement

The datasets presented in this study can be found in online repositories. The names of the repository/repositories and accession number(s) can be found in the article/Supplementary Material.

## Ethics Statement

The animal study was reviewed and approved by Institutional Biosafety Committee of Chulalongkorn University (IBC No. 2031002) and Queen Saovabha Memorial Institute Animal Care and Use Committee (Protocol No. QSMI-ACUC-01-2020). Written informed consent for participation was not obtained from the owners because this study was conducted on a remaining part of samples that routinely submitted and processed for rabies investigation.

## Author Contributions

CP and ST designed the study. SW and BW collected the samples. SW performed the experiments, analyzed the result, and wrote the first draft of the manuscript. TK, CP, and ST finalized the manuscript. All the authors approved the manuscript.

## Conflict of Interest

The authors declare that the research was conducted in the absence of any commercial or financial relationships that could be construed as a potential conflict of interest.

## Publisher's Note

All claims expressed in this article are solely those of the authors and do not necessarily represent those of their affiliated organizations, or those of the publisher, the editors and the reviewers. Any product that may be evaluated in this article, or claim that may be made by its manufacturer, is not guaranteed or endorsed by the publisher.
