# Supplementary material for: Molecular and Pathological Investigations of Selected Viral Neuropathogens in Rabies-Negative Brains of Cats and Dogs Revealed Neurotropism of Carnivore Protoparvovirus-1
Source: Front Vet Sci. 2021 Aug 19;8:710701. doi: 10.3389/fvets.2021.710701 (PMC8416986; doi:10.3389/fvets.2021.710701)
Supplement: Supplementary file 1 [file Table_1.DOCX]

**Molecular and pathological investigations of selected viral neuropathogens in rabies-negative brains of cats and dogs revealed neurotropism of carnivore protoparvovirus-1**

Sabrina Wahyu Wardhani^1,2^, Boonyakorn Wongsakul^3^, Tanit Kasantikul^4^, Chutchai Piewbang^2,5^, Somporn Techangamsuwan^2,5*^

**Supplementary Table S1** Sets of primer used in selective viral molecular screening in this study

| **Virus** | **Target gene^a^** | **Direction** | **Primer sequence 5’-3’** | **Target size (bp)** | **Reference** |
| --- | --- | --- | --- | --- | --- |
| *Coronaviridae* | ORF1b | Fwd | GGGTTGGGACTATCCTAAGTGTGA | 452 | Ksiazek et al., 2003 |
|  |  | Rev | TAACACACAACICCATCATCA |  |  |
| *Flaviviridae* | NS5 | Fwd | TGYRTBTAYAACATGATGGG | 269-272 | Cook et al., 2006 |
|  |  | Rev | GTGTCCCADCCDGCDGTRTC |  |  |
| *Herpesviridae* | DNA pol | Fwd (outer) | GAYTTYGCNAGYYTNTAYCC | 452-708 | VanDevanter et al., 1996 |
|  | (nested | Fwd (outer) | TCCTGGACAAGCAGCARNYSGCNMTNAA |  |  |
|  | PCR) | Rev (outer) | GTCTTGCTCACCAGNTCNACNCCYTT |  |  |
|  |  | Fwd (inner) | TGTAACTCGGTGTAYGGNTTYACNGGNGT | 228 |  |
|  |  | Rev (inner) | CACAGAGTCCGTRTCNCCRTADAT |  |  |
| *Paramyxoviridae* | RdRp | Fwd | GARGGIYIITGYCARAARNTNTGGAC | 100-150 | van Boheemen et al., 2012 |
|  |  | Rev | TIAYIGCWATIRIYTGRTTRTCNCC |  |  |
| *Protoparvovirus* | VP | Fwd | ATGGCACCTCCGGCAAAGA | 2246 | Mochizuki et al., 1996 |
|  |  | Rev | TTTCTAGGTGCTAGTTGAG |  |  |
| *Feline calicivirus* | VP | Fwd | GAACTACCCGCCAATCA | 120 | Piewbang et al, 2019 |
|  |  | Rev | AGCACRYCATATGCGGC |  |  |
| *Feline bocavirus* | VP | Fwd | AAAATCCTAAACAACAAA | 562 | Lau et al., 2012 |
|  |  | Rev | TATGGCAATTCTGGCATT |  |  |
| *Canine adenovirus* | E3 | Fwd | TATTCCAGACTCTTACCAAGAGG | 551 | Crawford-Miksza et al., 1999; Piewbang et al., 2017 |
|  |  | Rev | ATAGACAAGGTAGTARTGYTCAG |  |  |
| *Canine bocavirus* | NS1 | Fwd | AGGTCGGCCACTGGCTGT | 128 | Lau et al., 2012 |
|  |  | Rev | CAGCTTAACGGCATTCACTA |  |  |

^a^ ORF = Open reading frame; NS = Non-structural protein; DNA pol = DNA polymerase; RdRd = RNA-dependent RNA polymerase; VP = Viral protein; E3 = Early region 3.

**Supplementary Table S2** Primer sets used to obtain the complete genome of CPPV-1

| **Primer name** | **Primer sequences 5’-3’** | **Nucleotide position** | **Reference** |
| --- | --- | --- | --- |
| VPF | ATGGCACCTCCGGCAAAGA | 2285-2303 | Mochizuki et al., 1996 |
| VPR | TTTCTAGGTGCTAGTTGAG | 4530-4512 | Mochizuki et al., 1996 |
| FPLV_51 | CCAACTAAAAGAAGTAAACC | 2726-2745 | Mochizuki et al., 1996 |
| FPLV_9 | TCCTGCTGGATATCTTCCT | 4042-4060 | Mochizuki et al., 1996 |
| FPLV_23 | CTTTCCTCCAAAAATCTGA | 4397-4415 | Mochizuki et al., 1996 |
| FPLV_41 | ATTGTATACCATATAACAAACC | 4738-4759 | Mochizuki et al., 1996 |
| FPLV_8 | AATACAAACTATATTACTGAAG | 3785-3806 | Mochizuki et al., 1996 |
| FPLV_10 | TGTCATAAAGCCATGT | 3108-3125 | Mochizuki et al., 1996 |
| FPLV_1 | GTACATTTAAATATGCCAGA | 3029-3408 | Mochizuki et al., 1996 |
| NS Fext | GACCGTTACTGCATTCGCTTC | 206-227 | Pérez et al., 2014 |
| NS Rint | CATCATCCAGTCTTCAGGTG | 1167-1186 | Pérez et al., 2014 |
| NS Fint | GTTGAAACCACAGTGACGACAG | 1105-1076 | Pérez et al., 2014 |
| NS Rext | GAAGGGTTAGTTGGTTCTCC | 2441-2460 | Pérez et al., 2014 |

**Supplementary Table S3** Antibody and antigen retrieval method used for immunohistochemistry

| **Viruses** | **Antigen retrieval method** | **Primary antibody** | **Notes** |
| --- | --- | --- | --- |
| *Feline parvovirus* (FPV), *Canine parvovirus* (CPV) | 0.1% trypsin, 30 m | Monoclonal anti-canine parvovirus  1:200 dilution (Abcam, ab140431, UK) | Reacts with FPV, CPV, and Mink enteritis virus (MEV) |
| *Feline alphaherpesvirus* | Autoclave (121°C, 5 m), citrate buffer pH 6.0 | Monoclonal anti-Feline herpesvirus clones WIGH strain  1:500 dilution | Courtesy by Dr. Tsuchiya Katoro, Nippon Institute of Biological Science, Japan |
| *Feline infectious peritonitis virus* type 1 and type 2 | Autoclave (121°C, 10 m), citrate buffer pH 6.0 | Coronavirus pan Monoclonal Antibody (FIPV3-70)  1:100 dilution (Thermo, MA1-82189, USA) | Courtesy by Dr.Sukullaya Assarasakorn, Faculty of Veterinary Science, Chulalongkorn University, Thailand |
| *Feline calicivirus* | Autoclave (121°C, 10 m), citrate buffer pH 6.0 | Monoclonal anti-Feline calicivirus  1:100 dilution (Abcam, ab33990, UK) |  |
| *Canine distemper virus* | Autoclave (121°C, 10 m), distilled water | Monoclonal anti-Canine distemper virus  1:200 dilution (ViroStat, Portland, USA) |  |

**Supplementary Table S4** BLASTn analysis results of the partial VP2 gene sequences obtained from cats and dogs in this study

| **Case number** | **Sequence alignment result** |
| --- | --- |
| B31 | *Feline panleukopenia virus* (MN400978), 99.37% identity |
| B32 | *Feline panleukopenia virus* (KX434461), 99.58% identity |
| B34 | *Feline panleukopenia virus* (MN127781), 99.82% identity |
| B57 | *Feline panleukopenia virus* (MH669800), 99.89% identity |
| B59 | *Feline panleukopenia virus* (KX434461), 99.36% identity |
| B64 | *Feline panleukopenia virus* (MN127779), 99.86% identity |
| B69 | *Feline panleukopenia virus* (KP019621), 99.87% identity |
| B74 | *Feline panleukopenia virus* (AY665655), 99.34% identity |
| B75 | *Feline panleukopenia virus* (MN127781), 99.44% identity |
| B82 | *Feline panleukopenia virus* (MN127779), 99.91% identity |
| B86 | *Feline panleukopenia virus* (MH669800), 99.38% identity |
| B88 | *Feline panleukopenia virus* (MN127779), 100% identity |
| B94 | *Feline panleukopenia virus* (AY665655), 99.02% identity |
| B127 | *Feline panleukopenia virus* (MH669800), 99.18% identity |
| B63 | *Canine parvovirus* (MK388674), 99.46% identity |
| B90 | *Canine parvovirus* (MT010564), 99.77% identity |
| B102 | *Canine parvovirus* (MT010564), 99.93% identity |
| B110 | *Canine parvovirus* (MK518015), 99.09% identity |
| B111 | *Canine parvovirus* (MK518015), 99.66% identity |
| B112 | *Canine parvovirus* (MT010564), 99.63% identity |

**Supplementary Table S5** Complete genome sequences to construct phylogenetic tree including sequences obtained in this study and sequences retrieved from GenBank

| **Accession no.**  **(Sequence name in this study)** | **Origin** | **Year collected** | **Genotype** |
| --- | --- | --- | --- |
| MW589466  (CPV 2c/TRC-B63/Thailand/2020) | Thailand | 2020 | CPV-2c |
| MW589467  (CPV 2c/TRC-B102/Thailand/2020) | Thailand | 2020 | CPV-2c |
| MW589468  (CPV 2c/TRC-B90/Thailand/2020) | Thailand | 2020 | CPV-2c |
| MW589469  (CPV 2c/TRC-B112/Thailand/2020) | Thailand | 2020 | CPV-2c |
| MW589470  (CPV 2c/TRC-B111/Thailand/2020) | Thailand | 2020 | CPV-2c |
| MW589471  (FPV/TRC-B34/Thailand/2020) | Thailand | 2020 | FPV |
| MW589472  (FPV/TRC-B88/Thailand/2020) | Thailand | 2020 | FPV |
| MW589473  (FPV/TRC-B59/Thailand/2020) | Thailand | 2020 | FPV |
| MH545963.1 | India | 2018 | CPV-2a |
| KR002805.1 | China | 2014 | CPV-2a |
| KR002792.1 | China | 2013 | CPV-2a |
| KR002803.1 | China | 2014 | CPV-2a |
| KR002797.1 | China | 2013 | CPV-2a |
| KR002794.1 | China | 2013 | CPV-2a |
| KR002801.1 | China | 2014 | CPV-2a |
| KR002798.1 | China | 2013 | CPV-2a |
| KR002795.1 | China | 2013 | CPV-2a |
| KR002804.1 | China | 2014 | CPV-2a |
| KR002802.1 | China | 2014 | CPV-2a |
| KR002800.1 | China | 2014 | CPV-2a |
| KM457141.1 | Uruguay | 2011 | CPV-2a |
| KM457138.1 | Uruguay | 2011 | CPV-2a |
| KM457140.1 | Uruguay | 2011 | CPV-2a |
| KR002799.1 | China | 2013 | CPV-2b |
| KR002796.1 | China | 2013 | CPV-2b |
| KR002793.1 | China | 2013 | CPV-2b |
| JQ268284.1 | China | 2011 | CPV-2b |
| MN832850.1 | Taiwan | 2018 | CPV-2c |
| MH711902.1 | Thailand | 2016 | CPV-2c |
| MH711894.1 | Thailand | 2016 | CPV-2c |
| KM457129.1 | Uruguay | 2011 | CPV-2c |
| KM457142.1 | Uruguay | 2011 | CPV-2c |
| KM457130.1 | Uruguay | 2011 | CPV-2c |
| KM457131.1 | Uruguay | 2011 | CPV-2c |
| KY073269.1 | Brazil | 2015 | CPV-2c |
| MN451692.1 | USA | 2011 | FPV |
| MF069445.1 | Columbia | 2015 | FPV |
| MN451652.1 | Finland | 1983 | FPV |
| MN127779.1 | Thailand | 2018 | FPV |
| MN127780.1 | Thailand | 2019 | FPV |
| MN127781.1 | Thailand | 2019 | FPV |
| MG764511.1 | China | 2015 | FPV |
| KX685354.1 | China | 2016 | FPV |
| MG924893.1 | China | 2016 | FPV |
| KP280068.1 | China | 2014 | FPV |
| MH559110.1 | India | 2018 | FPV |
| KT899746.1 | China | 2010 | MEV |
| KT899745.1 | China | 2014 | MEV |
| KT899746.1 | China | 2010 | MEV |

**Supplementary Table S6** Summary of the histopathological changes and immunohistochemical (IHC) analysis on the representative sections of positive PCR samples

| **Sample no.** | **cPCR/RT-PCR** | **Histopathological changes** | **IHC** |
| --- | --- | --- | --- |
| B29 | FCV | No significant change | Strong immunoreactivity in cerebral neurons |
| B135 | FCV | No significant change | Weak immunoreactivity in large neurons of brain stem |
| B100 | FCoV and FeHV-1 | Moderate non-suppurative meningitis | Strong immunoreactivity of FCoV and FeHV-1 antigens in astrocytes and inflammatory cell in the area of meningitis, respectively. |
| B31 | FPV | Mild gliosis | Strong immunoreactivity in cerebral neurons |
| B32 | FPV | Severe non-suppurative encephalitis with 2-3 layer of lymphocytic perivascular cuffing, moderate gliosis, mild neuronal necrosis and neuronophagia | Strong immunoreactivity in cerebral neurons and large neurons of brain stem |
| B34 | FPV | Mild non-suppurative meningitis with the addition of mild gliosis, neuronal necrosis, and focal necrosis | Strong immunoreactivity in cerebral neurons |
| B64 | FPV | No significant change | Strong immunoreactivity in Purkinje neurons |
| B110 | CPV-2c | No significant change | Weak immunoreactivity in cerebral neurons |
| B111 | CPV-2c | Mild autolysis, no significant change | Weak immunoreactivity in cerebral neurons |
| B112 | CPV-2c | No significant change | Strong immunoreactivity in cerebral neurons |
| B118 | CDV Asia-4 | Mild non-suppurative encephalitis with mild lymphocytic perivascular cuffing, gliosis, and neuronal necrosis | Strong immunoreactivity in cells composing cerebellar molecular layer |
| B132 | CDV Asia-4 | Moderate autolysis, no significant change | Strong immunoreactivity in astrocytes |
| B137 | CDV Asia-4 | Mild satellitosis | Strong immunoreactivity in astrocytes |

**
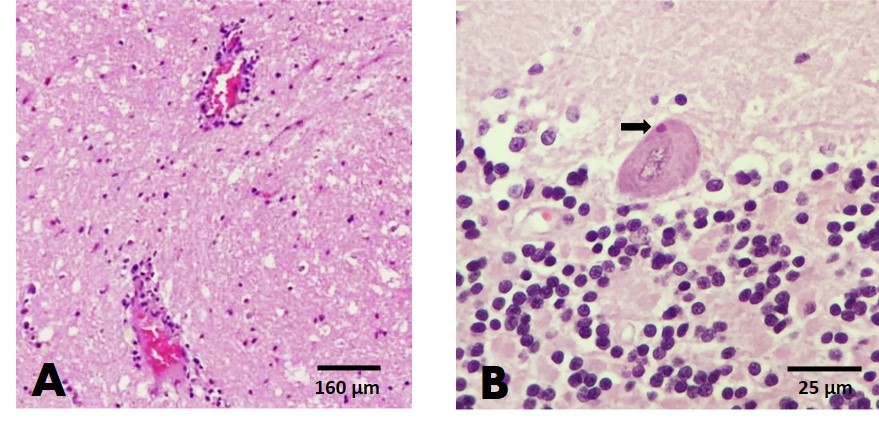
**

**Supplementary Figure S1.** Brain, dog. H&E section revealed perivascular cuffing composed of predominantly lymphocytes (**A,** 100x) and intracytoplasmic inclusion body (arrow) (**B,** 400x) in the cerebellum of dog in which specific pathogen was failed to be detected by PCR panels in this study.
